# Genomic analysis of prophage prevalence and diversity in the bovine mastitis pathogen Streptococcus uberis

**DOI:** 10.1099/mgen.0.001849

**Published:** 2026-09-28

**Authors:** Thomas Smith-Zaitlik, Logan L. Newstead, Katrina Henderson, Colin Mason, Nuno Silva, Geoffrey Foster, Alison S. Low, Alastair Macrae, David L. Gally, Gavin K. Paterson

**Affiliations:** 1 Royal (Dick) School of Veterinary Studies and the Roslin Institute, University of Edinburgh, Edinburgh, UK; 2 SRUC Veterinary Services, Dumfries, UK; 3 Department of Disease Control, Moredun Research Institute, Edinburgh, UK; 4 SRUC Veterinary Services, Inverness, UK; 5 The Roslin Institute and Royal (Dick) School of Veterinary Studies, University of Edinburgh, Edinburgh, UK

**Keywords:** bacterial genome, bacteriophages, bovine mastitis, streptococcal phages, *Streptococcus uberis*, veterinary medicine

## Abstract

The prevalence and diversity of prophages carried by the major global bovine mastitis pathogen, *Streptococcus uberis*, are poorly characterized. Bacteriophages (phages) are viruses which can infect and kill bacteria. Prophages, phages that have integrated into the bacterial genome, can have a profound impact on the fitness of their host cell. Here, we analysed the prophage content of 661 *S*. *uberis* genomes, including 345 newly sequenced isolates. Using PhiSpy, we identified 766 prophages, which formed 2 distinct clades postulated to correspond to full-length and satellite prophages, respectively. Putative full-length prophages had larger genomes and encoded more structural and lytic genes. Putative satellite prophages, which are reliant on helper phages for their replication and lysis, had smaller genomes, fewer structural genes and no lysins. Genomic and phylogenetic differences between the clades suggested that these satellite prophages were distinct from the full-length prophages, rather than being cryptic remnants of the latter. We identified a diverse repertoire of prophage-encoded endolysins with potential as novel therapeutics. Finally, we contextualized our *S. uberis* prophages within the broader phylogeny of *Streptococcus* phages, by aligning them with 1,078 publicly available phage genomes. The same division between putative full-length and satellite phages was maintained in this larger *Streptococcus* phage phylogeny of 1,844 phages. Our findings underline the prevalence and diversity of prophages within *S. uberis* and lay the foundations for furthering our understanding of phage–host interactions in this significant veterinary pathogen.

**Impact Statement**

Bacteriophages (phages) are viruses which can infect and kill bacteria, and prophages are phages that have integrated into the bacterial genome and can affect the properties of their host bacterium. This study searched for prophages in a large collection of *Streptococcus uberis* genomes, an important bovine mastitis pathogen. We identified a diverse population of *S. uberis* prophages and placed these into a wider context through their comparison with publicly available streptococcal phages to provide a genus-wide phage analysis. Additionally, we highlight the diversity of prophage endolysins within *S. uberis* which could be investigated as novel therapeutics. Investigation of phage–bacterium interactions in *S. uberis* may provide a better understanding of the genomic diversity and disease pathogenesis of this important veterinary pathogen.

## Data Summary

Metadata and accessions for all sequenced isolates studied are provided in Table S1 with the 345 newly reported sequenced isolates being deposited in BioProject PRJNA1064901.

## Introduction

*Streptococcus uberis* is a Gram-positive bacterium and a leading cause of bovine mastitis worldwide [1]. While the disease pathogenesis of *S. uberis* is not well understood, infections can originate from environmental sources such as contaminated bedding [2–5], or via contagious spread between cows [3–6]. Bovine mastitis is inflammation of the mammary gland which can be caused by infection or physical trauma. Infections can be clinical or sub-clinical, with the latter often becoming chronic [7, 8]. Bovine mastitis causes major economic losses, both directly through treatment and labour costs, but also indirectly, due to lower milk production and quality [9, 10]. In Great Britain between 2017 and 2024, *S. uberis* was isolated from 19.4% of bovine mastitis cases making it the second most common organism isolated [11].

Bacteriophages (phages) are viruses which can infect and kill bacteria. Following successful infection, obligately lytic phages replicate and lyse the infected cell, allowing the release and dissemination of progeny phage. Prophages can integrate into the bacterial genome or exist in the cytoplasm as an episomal element [12]. Under certain conditions, particularly those causing DNA damage, prophages are induced to enter the lytic cycle, lysing the host cell in the process. Prophages can contribute to horizontal gene transfer (referred to as transduction), by encoding accessory genes (i.e. those not required for phage replication); they can confer improved fitness on their host bacterium, such as antimicrobial resistance [13] or auxiliary metabolic activity [14]. Cryptic and satellite prophages lack the genes required to independently form a phage virion. Satellite prophages are therefore reliant on hijacking the necessary genes from intact ‘helper’ phages [15, 16].

Few studies have reported on the prophage content of *S. uberis*, and often these have focused on only a small number of isolates. Lysogenic phages can be readily induced from clinical *S. uberis* isolates (in 38 out of 98 strains tested), but beyond their potential for use in strain typing, these were not further characterized [17]. Whilst bioinformatic analyses of whole-genome-sequenced isolates have identified intact prophages in mastitis *S. uberis* isolates from Australia (9/27; 33.33%) [18], Bangladesh (2/5; 40.00%) [19], Germany (12/24; 50.00%) [20], the UK (7/12; 58.33%) [21] and Thailand (94/138; 68.11%) [22], little further analysis has been done. Other studies also note the presence of incomplete or satellite prophages within *S. uberis* genomes [15, 19–21]. Limited data are available on how prophages might influence *S. uberis* host strain behaviour or fitness, for example, through the carriage of AMR, virulence or metabolism-associated genes. Prophages are also of interest in the search for novel antimicrobials, such as prophage-derived endolysins, with several already shown to have activity against *S. uberis*. For example, *Streptococcus agalactiae* prophage endolysin B30 [23] showed strong killing efficacy against *S. uberis* in milk *in vitro* [24]. Two prophage endolysins isolated from *Streptococcus suis*, PlySs2 and PlySs9, also showed *in vitro* killing activity against *S. uberis* bovine mastitis isolates [25]. The endolysin Ply700, identified from an *S. uberis* prophage following mitomycin C treatment, shows *in vitro* activity against *S. uberis*, *Streptococcus pyogenes* and *Streptococcus dysgalactiae* [26].

We report on the identification of 766 prophages from 661 *S*. *uberis* genomes, to our knowledge, the largest prophage study of the species to date. A comparison of our prophages to 1,078 publicly available streptococcal phages and satellite prophages provides a genus-wide *Streptococcus* phage phylogeny which highlights the distinction between full-length and satellite prophages. Finally, we identify a diverse repertoire of prophage-encoded endolysins with translational potential as novel therapeutics against this prevalent veterinary pathogen.

## Methods

### *S. uberis* isolates

Draft genome assemblies were generated for this study from 345 *S*. *uberis* bovine mastitis isolates collected by routine diagnostic work by SRUC Veterinary Services, Moredun Research Institute and Dairy Herd Health and Productivity Services [Royal (Dick) School of Veterinary Studies, University of Edinburgh]. Whole-genome Illumina NovaSeq sequencing was performed by MicrobesNG (University of Birmingham, Birmingham, UK). Sequenced isolates from this study were supplemented with 316 publicly available *S. uberis* genomes for a total of 661 genomes (Table S1, available in the online Supplementary Material). Raw reads were trimmed using Trimmomatic (Version 0.39); quality was assessed using FastQC (Version 2.1.11) (https://github.com/s-andrews/FastQC) and multiQC (Version 1.13) [27]. Assembly was performed using SPAdes (Version 3.15.5) [28]. Accessions and assembly quality data, generated by Quast [29], for all sequenced isolates are provided in Table S1, and those newly reported in this study are in BioProject PRJNA1064901.

### Bacterial phylogeny

*S. uberis* genomes were analysed using Snippy (Version 4.6.0) [30], using *S. uberis* 0140J as a reference genome. Tools were used on default settings unless otherwise stated. A core genome alignment file was then generated and cleaned using Snippy-core and Snippy-clean, respectively. Gubbins (Version 3.3) [31] and SNP-Sites (Version 2.5.1) [32] were then used to reconstruct core alignments to reduce the impact of regions of recombination. Gubbins was run using the FastTree tree-builder option, and SNP-Sites was run using the –c flag. IQTree (Version 3.0.1) [33] was used to generate a tree file from the core genome alignment using the GTR substitution model and 1,000 replicates.

### Prophage identification and quality control

Putative prophages were identified in the 661 *S*. *uberis* genomes using PhiSpy (Version 4.2.21) [34] on default settings. Quality and completeness of putative prophages were assessed using CheckV (Version 1.0.1) on default settings [35]. Prophages with a quality score of medium or better were retained for analysis (Table S2). An unpaired two-sample Wilcoxon test was used to compare the number of prophages in USA and non-USA *S. uberis* isolates. Statistical analysis was performed in RStudio (Version 4.2.2) [36].

### Prophage annotation

Prophage sequences were annotated using Prokka (Version 1.14.6) [37] using the compliant flag and with the prokaryotic virus remote homologous group (PHROG) dataset added [38]. Annotations were manually classified using categories provided by the National Center for Biotechnology Information (NCBI) database of clusters of orthologous genes (COG) (https://www.ncbi.nlm.nih.gov/research/cog) [39] and the PHROG annotation table (https://phrogs.lmge.uca.fr/). Using these categories, annotations were classified as follows: hypothetical; replication; structural; moron, auxiliary metabolic and host takeover; tRNA; or lysis genes.

Pangenome analysis was performed using Roary (Version 3.13.0) [40] on default settings. The pangenome-clustered protein file was analysed for two sets, to compare pangenomes for *S. uberis* prophage sequences from blue (putative full-length prophages) and green clades (putative satellite prophages), respectively. The number of unique and shared genes in each group was visualized using ggplot2 (Version 3.4.2) [41] in RStudio (Version 4.2.2) [36].

### Endolysin analysis

Nucleotide sequences for prophage genes annotated as endolysins were extracted and aligned using Clustal-Omega (Version 1.2.4) [42] with –distmat-out, –full, and –iter=5 options. IQTree (Version 3.0.1) was used to generate a tree file from the alignment with 1,000 replicates [33]. Nucleotide alignments were visualized using the ggTree package [43] in RStudio (Version 4.2.2) [39]. Phytools (Version 2.3.0) [44] was used to visualize co-phylogenetic trees. Nucleotide sequences for four characterized streptococcal phage endolysins (B30, AY149214.3 [23]; PlySs2, AGF87539.1 [25]; PlySs9, AGF87618.1 [25]; and Ply700, ABB02702.1 [26]) were included for comparison. These were aligned with prophage endolysins as described. Percentage identity was calculated using the –percent-id flag when generating the distance matrix.

### Phage phylogeny and protein cluster mapping

Streptococcal phages (Table S3) were downloaded from the Virus-Host Database (VHDB) [45, 46]. This dataset included both lytic and lysogenic phages. Streptococcal satellite prophages (Table S3) identified by Javan *et al.* [15] were downloaded from NCBI [39]. ViPTreeGen (Version 1.1.3) (https://github.com/yosuken/ViPTreeGen) was run on default settings to generate a proteomic alignment for *S. uberis* prophages (Table S4) from this study and publicly available streptococcal phages (Table S5), respectively. Alignments were visualized using the ggTree (Version 3.6.2) package [43] in RStudio (Version 4.2.2) [36].

vContact2 (Version 0.11.3) was run with --db ‘None’ option and used to generate gene-sharing networks using protein clusters shared between phage genomes [47]. Cytoscape was used to visualize a network for *S. uberis* prophages, 730 streptococcal phages from the VHDB and 341 satellite prophages [15] with duplicate edges removed.

## Results

### Prevalence of prophages within *S. uberis*

Using PhiSpy, we analysed 661 *S*. *uberis* genomes from 6 countries and identified 1,054 putative prophage regions. CheckV scoring was used as a quality filter, and all putative prophages with a quality score of medium or higher were kept for downstream analyses. CheckV annotates host and viral genes using HMMs to identify bacterial contamination, estimates completeness against known viral genomes, predicts repeat regions to identify closed genomes and summarizes these findings as a quality score. This removed 288 putative prophage regions from the dataset, leaving 766 prophages for analysis. A core genome alignment of host *S. uberis* strains was visualized alongside the country of origin and prophage count (Fig. 1). The majority of *S. uberis* strains analysed (547/661; 82.75%) carried at least one prophage. We identified between 0 and 3 prophages per strain, with a mean of 1.16 prophages per genome. However, this was much lower (mean of 0.3 prophages per genome) in USA isolates (Fig. 1, black bar) which were closely related to one another. The number of prophages identified in non-USA *S. uberis* strains was significantly greater than those from the USA $\left(p=8.40\times 10^{-15}\right)$.

**Fig. 1.**
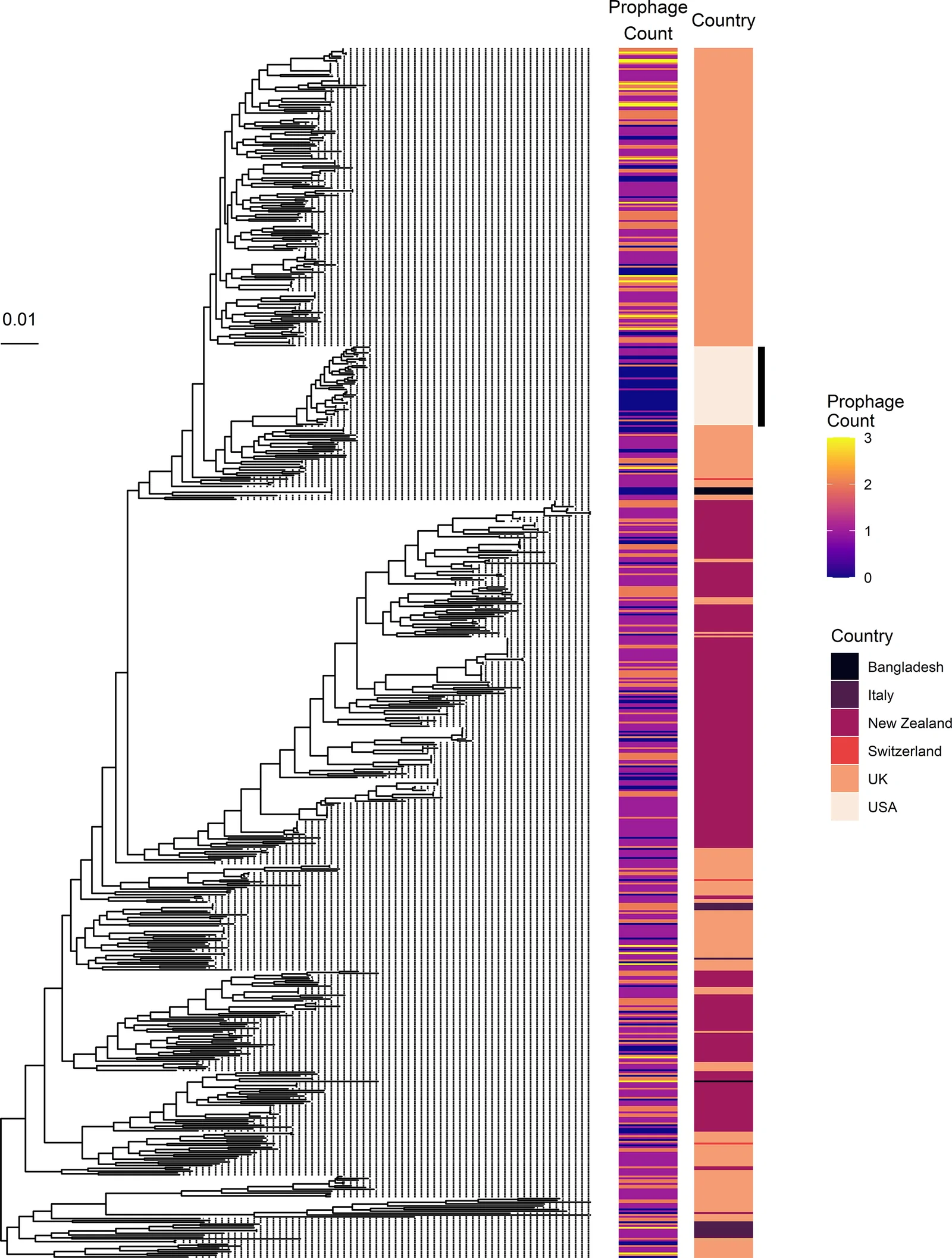
Phylogenetic tree of *S. uberis* (*n*=661) draft genomes and the number of prophages identified in each host strain and the country of origin. Alignment was generated using Snippy, Gubbins and IQTree (replicates *n*=1,000). The tree was visualized using ggTree in RStudio. The black bar denotes region of low numbers of prophages carried in USA isolates.

### Full-length prophages are phylogenetically distinct from satellite prophages in *S. uberis*

Proteomic alignment of 766 *S*. *uberis* prophages highlighted 2 distinct clades (Fig. 2). Prophages in the blue clade had larger genomes and a higher G+C content compared with those in the green clade. Additionally, the mean average number of structural gene annotations was 12.05 and 0.23 for the blue and green clades, respectively. A smaller genome, as well as reduced G+C content, and a lack of structural genes suggested that prophages within the green clade were likely to be cryptic or satellite prophages (Fig. 2). Satellite prophages often require a helper phage to successfully replicate and escape the host cell, whereas larger, higher G+C content prophages encoding structural genes are more likely to be complete or full-length prophages, capable of independent excision and replication. In total, there were 448 putative full-length and 318 putative satellite prophages. Full-length prophages were present in over half (349/661; 52.80%) of *S. uberis* isolates, with satellite prophages present in (277/661; 41.91%). Furthermore, consistent with the division of the blue and green clades into full-length and satellite prophages, respectively, all 259 endolysin and 363 holin genes annotated belonged to prophages in the full-length, blue clade.

**Fig. 2.**
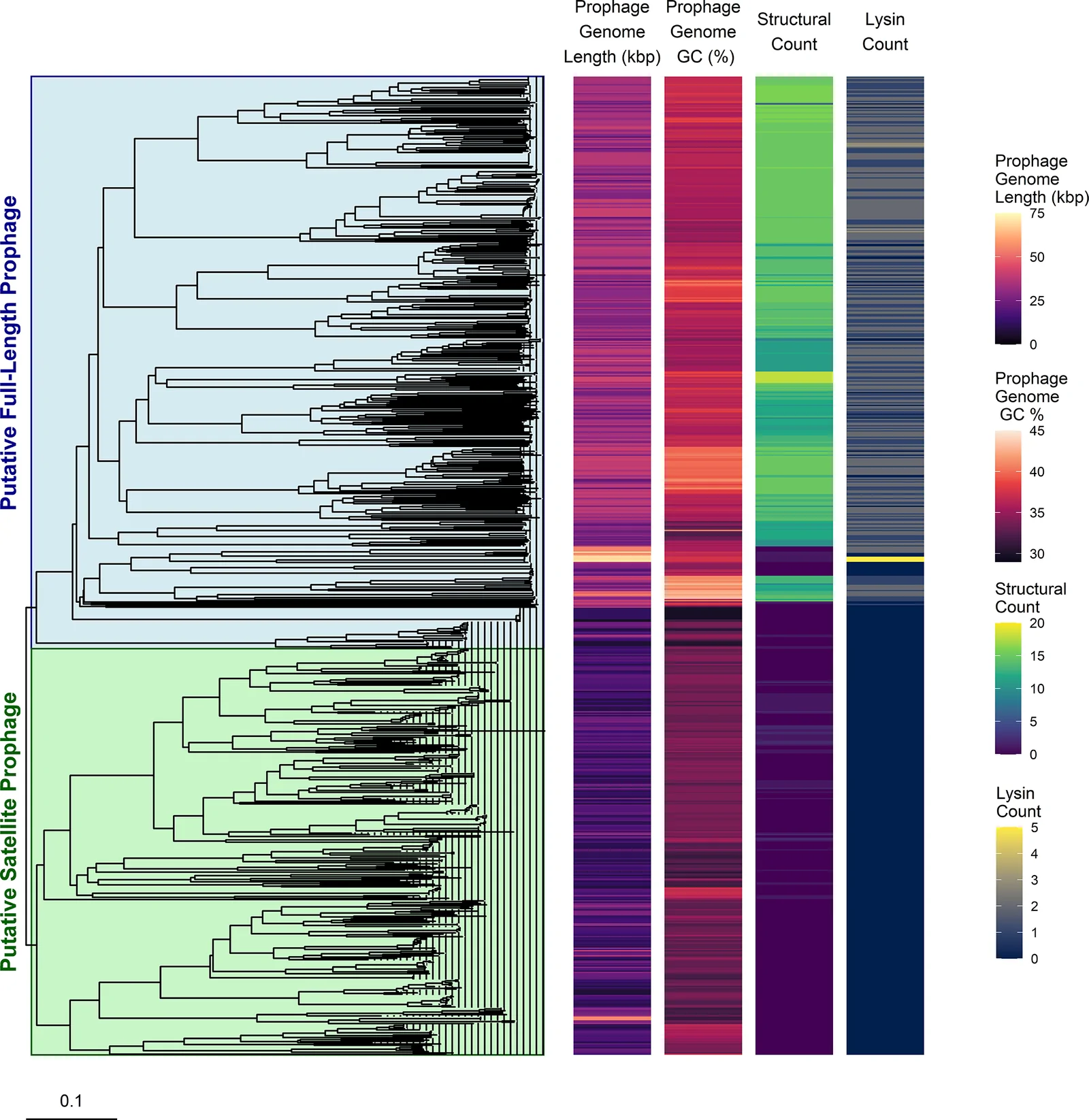
Proteomic alignment of *S. uberis* prophage (*n*=766) genomes, genome length kbp, G+C content (mol%) and the number of structural and lysin (holin+endolysin) gene annotations. Two clades are highlighted in blue, designated as putative full-length prophage (*n*=448), and green, designated as putative satellite prophages (*n*=318). The tree was visualized using ggTree in RStudio.

### Full-length and satellite prophages have separate pangenomes

Roary pangenome analysis was used to compare differences in gene content between full-length (blue clade) and satellite prophages (green clade). A total of 4,606 unique genes were identified in the *S. uberis* prophage pangenome (Fig. 3a). Most genes (3,606/4,606; 78.23%) in the *S. uberis* prophage pangenome were unique to full-length prophages, with 971 (21.08%) unique to satellite prophages. Very few genes (29/4,606; 0.63%) were shared between full-length and satellite prophages (Fig. 3a). The most common annotation category was ‘hypothetical protein’ across the prophage pangenome (2,830/4,606; 61.44%) and within the genes unique to the full-length (2,110/3,606; 58.51%), satellite (702/971; 72.30%) and shared (18/29; 62.07%) groups.

**Fig. 3.**
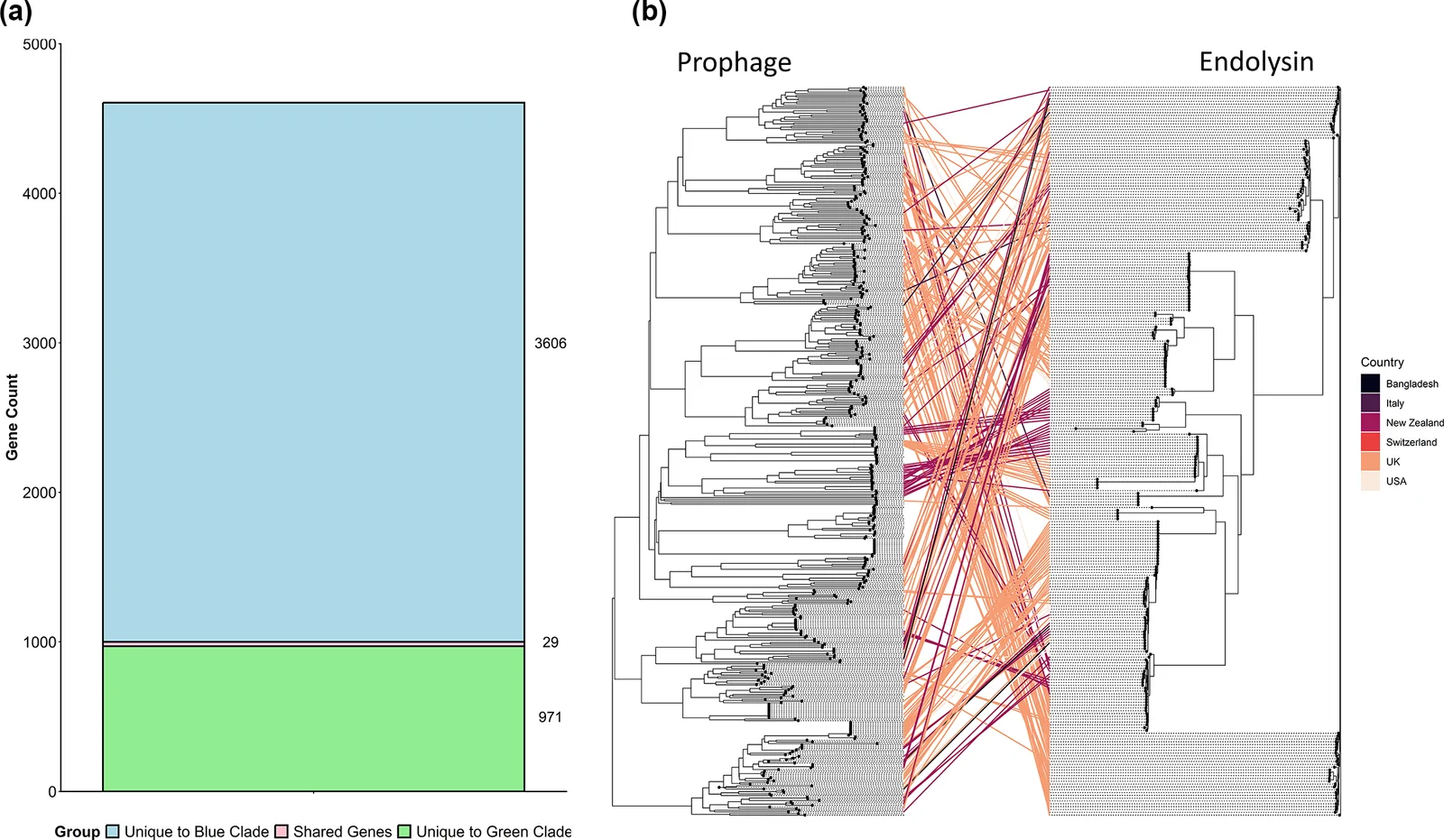
Comparison of *S. uberis* prophage gene content. (a) Roary pangenome comparison of prophage gene content in putative full-length prophages (blue clade, Fig. 1) (*n*=448) and putative satellite prophages (green clade, Fig. 1) (*n*=318). (b) Co-phylogenetic tree for blue clade prophage proteomic alignment and endolysin Clustal Omega tree; line colour corresponds to the country of host strain origin. Trees were visualized using Phytools in RStudio.

However, in the other types of unique genes present, differences were observed between full-length and satellite prophages. Full-length prophages encoded a greater proportion of unique structural genes (609/3,606; 16.89% of pangenome genes) such as tail fibres and head proteins compared to satellite prophages (4/971; 0.41%). Satellite prophages had a greater proportion of unique replication-related genes (226/971; 23.27%) such as integrases, compared to full-length prophages (627/3,606; 17.39%). This may simply reflect their comparative lack of structural gene annotations. Four of the 29 prophage pangenome genes shared between full-length and satellite prophages were classified as structural (head-tail adaptor Ad1, head closure Hc1, minor head protein and portal protein). The lack of shared genes between full-length (blue clade) and satellite prophages (green clade) highlights their genetic distinction, consistent with the latter being genuine satellite prophages rather than cryptic relatives of the full-length prophages.

Prokka annotated 259 endolysins and 363 holins, but these were only identified in full-length prophages (Fig. 3b). This further supports the proposal that prophages within the green clade (Fig. 2) are satellite phages, lacking the genes required to independently complete a lytic lifecycle.

Some highly conserved endolysins were found in disparately related prophages, such as those at the top of the endolysin tree (Fig. 3b), whereas some endolysins were highly conserved amongst closely related prophages, particularly those identified in *S. uberis* isolates from New Zealand.

### Full-length *S. uberis* prophages encode a diverse repertoire of endolysins

We aligned all 259 *S*. *uberis* prophage endolysin genes from the present study and included four characterized streptococcal endolysins shown to have activity against *S. uberis* [23, 25, 26]. Endolysins were selected from the literature for their efficacy against *S. uberis* but also their diversity from one another (≤35.30% nucleotide identity). This revealed a diverse repertoire of prophage-encoded endolysins (Fig. 4a) with the majority of the endolysins sharing little similarity with the previously characterized endolysins. The closest relatives from the current study to the four characterized endolysins were determined from the percentage identity matrix and visualized in closer detail. The characterized endolysin PlySs9 was most closely related to the endolysin from vB_SubZ_Chill (96.19% nucleotide identity), which was isolated from a clinical bovine mastitis case in Scotland (Fig. 4b). The closest relative to the previously studied Ply700 endolysin was encoded by vB_SubZ_Post (90.86% nucleotide identity), isolated from a clinical bovine mastitis case in the USA (Fig. 4c). Additionally, two other endolysins had >90% identity to Ply700. In contrast, previously characterized endolysins PlySs2 and B30 were not highly related to endolysins from this study (Fig. 4b, c) with a nucleotide identity to their nearest match of 28.73% and 41.39%, respectively.

**Fig. 4.**
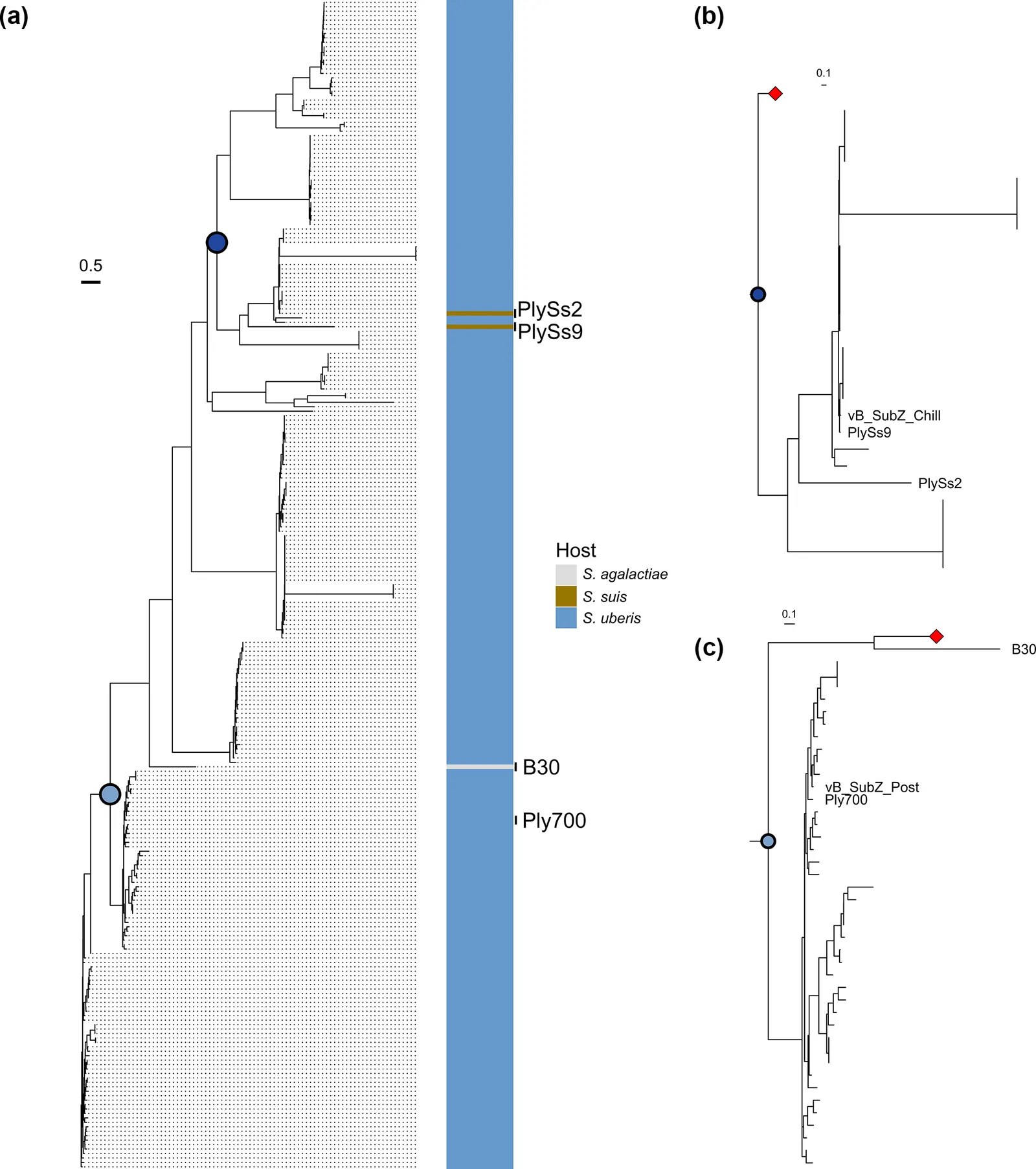
Clustal Omega alignment of streptococcal bacteriophage endolysins (*n*=263) and their host species. (**a) **Four publicly available endolysin sequences were aligned with *S. uberis* endolysins from the present study (*n*=259). The clades for (**b) **PlySs2 and PlySs9 and the clades for (**c) **B30 and Ply700 were shown in greater detail. Dark blue and light blue circles were used to represent the start of clades shown in panels b and c, respectively. Red diamonds represented collapsed nodes. Alignment was generated using Clustal Omega and IQTree (replicates *n*=1,000). The tree was visualized using ggTree in RStudio.

### Phylogenetic and protein cluster mapping comparison with other streptococcal phages

To understand the position of *S. uberis* prophages within the broader phylogeny of *Streptococcus* phages, we aligned them with 730 lytic and lysogenic streptococcal phages from the VHDB and 348 satellite prophages from Javan *et al*. [15]. The 1,844 phages in this dataset originated from 55 different *Streptococcus* species, with *S. uberis* the most frequent (786/1,844; 42.62%), largely due to the 766 phages contributed by the current study.

Sixteen *S. uberis* prophages from the current study had high genomic similarity (SG) to 22 publicly available *Streptococcus* phages (Table 1). Several full-length prophages showed similarity not only to those from the VHDB which came from *S. uberis*, but also to phages from other *Streptococcus* species, whereas satellite prophages from the present study were closely related only to other *S. uberis* satellite phages.

**Table 1.** Study *S. uberis* prophages with high similarity (SG) to VHDB and Javan *et al*. (2019) satellite phages. SG score rounded to two decimal places

Study prophage	Literature phage	GenBank	Host	SG score	Source
vB_SubZ_Freddy	*Streptococcus* phage Javan 620	MK448581	*S. uberis*	1	[15] satellite prophage
vB_SubZ_Nice	*Streptococcus* phage Javan 620	MK448581	*S. uberis*	1	[15] satellite prophage
vB_SubZ_Field	*Streptococcus* phage Javan 620	MK448581	*S. uberis*	1	[15] satellite prophage
vB_SubZ_Maple	*Streptococcus* phage Javan 625	MK448585	*S. uberis*	1	[15] satellite prophage
vB_SubZ_Clear	*Streptococcus* phage Javan 618	MK448994	*S. uberis*	1	VHDB
vB_SubZ_Clear	*Streptococcus* phage Javan 405	MK448752	*Streptococcus parauberis*	0.98	VHDB
vB_SubZ_Milk	*Streptococcus* phage Javan 620	MK448581	*S. uberis*	0.97	[15] satellite prophage
vB_SubZ_Red	*Streptococcus* phage phi29854	MT311967	*S. pyogenes*	0.96	VHDB
vB_SubZ_Honour	*Streptococcus* phage Javan 95	MK448836	*Streptococcus canis*	0.93	VHDB
vB_SubZ_Honour	*Streptococcus* phage Javan 639	MK448825	*Streptococcus urinalis*	0.93	VHDB
vB_SubZ_Red	*Streptococcus* phage Javan 244	MK448883	*Streptococcus gordonii*	0.92	VHDB
vB_SubZ_Muddy	*Streptococcus* phage Javan 635	MK448591	*S. uberis*	0.92	[15] satellite prophage
vB_SubZ_Red	*Streptococcus* phage phi29961	MT311968	*S. pyogenes*	0.92	VHDB
vB_SubZ_Dwarf	*Streptococcus* phage Javan 626	MK448995	*S. uberis*	0.91	VHDB
vB_SubZ_Rise	*Streptococcus* phage Javan 635	MK448591	*S. uberis*	0.91	[15] satellite prophage
vB_SubZ_Red	*Streptococcus* phage phi1207.3	AY657002	*S. pyogenes*	0.91	VHDB
vB_SubZ_Scent	*Streptococcus* phage Javan 635	MK448591	*S. uberis*	0.91	[15] satellite prophage
vB_SubZ_Red	*Streptococcus* phage phi29862	MT303952	*S. pyogenes*	0.91	VHDB
vB_SubZ_Comb	*Streptococcus* phage Javan 244	MK448883	*S. gordonii*	0.9	VHDB
vB_SubZ_Red	*Streptococcus* phage Javan 159	MK448687	*S. dysgalactiae*	0.9	VHDB
vB_SubZ_Bliss	*Streptococcus* phage Javan 244	MK448883	*S. gordonii*	0.9	VHDB
vB_SubZ_Gavin	*Streptococcus* phage Javan 623	MK448821	*S. uberis*	0.9	VHDB

Satellite prophage Javan 620 (MK448581) had previously been identified in *S. uberis* strain 0140J [15]. Our screening of the 0140J genome identified a satellite prophage, named vB_SubZ_Freddy, in the same location with 100% nucleotide identity to Javan 620. However, vB_SubZ_Freddy had a larger genome (19.09 kbp) than Javan 620 (12.22 kbp), meaning the two prophages only shared 64% coverage. Thus, these are the same single phage, at the same location in the same host strain but with a discrepancy over the length of the prophage.

Javan 620 was also closely related to prophages vB_SubZ_Nice, _Field and _Milk. The host strains for vB_SubZ_Freddy, Nice and Field were all isolated in the UK from bovine mastitis, whereas vB_SubZ_Milk was identified in an Italian ovine *S. uberis* strain.

The *Streptococcus* genus-wide phage phylogeny duplicated the division seen with *S. uberis* prophages of there being two clades which correlated with genome length, G+C content and the presence of structural genes (Fig. 5). Prophages in the top half of the phylogeny had longer average genome lengths (35.98 kbp) and higher G+C content (38.3 mol%), compared with prophages in the bottom half (14.21 kbp and 34.93%, respectively). Interestingly, prophages associated from some species, such as *Streptococcus thermophilus*, were concentrated within a small number of clades, whereas others, such as those from *S*. *pyogenes*, were distributed throughout the tree.

**Fig. 5.**
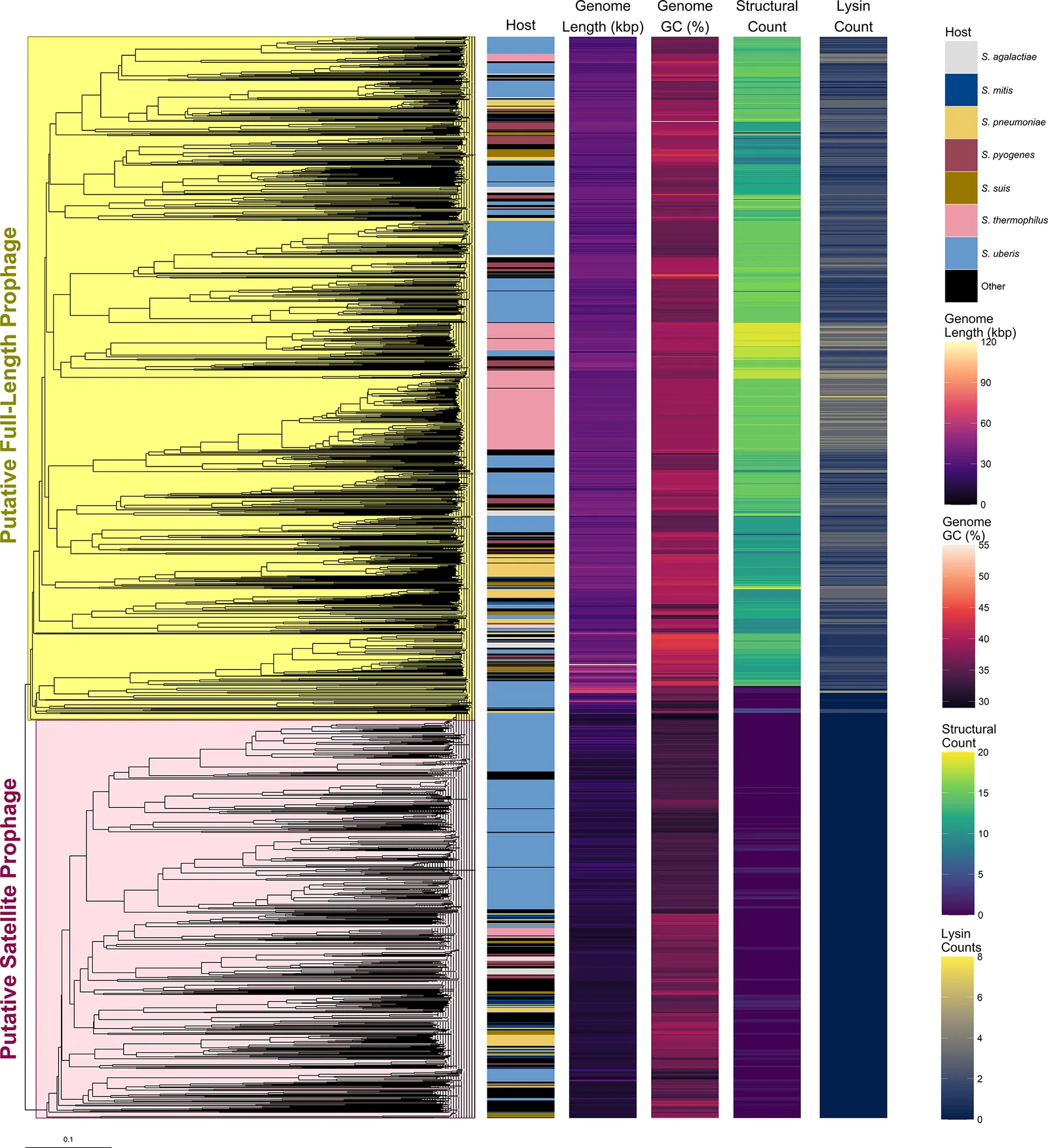
Proteomic alignment of study *S. uberis* prophages (*n*=766), Javan *et al.* [15] satellite prophages (*n*=348) and VHDB streptococcal phages (*n*=730) aligned with their genome length kbp, genomic G+C content (mol%) and structural and lysin (holin+endolysin) gene count. The seven most common hosts were assigned colours, and all other hosts were displayed as other. The pink clade denotes smaller genome and low genomic G+C content sequences. The tree was visualized using ggTree in RStudio.

While full-length *S. uberis* prophages were present in many different clades (Fig. 5, yellow clade), *S. uberis* satellite prophages typically clustered together (Fig. 5, pink clade). The satellite prophages present in the green clade (Fig. 2) were represented within the pink clade (Fig. 5). Satellite prophages identified by Javan *et al.* [15] also clustered within the pink clade. However, the presence of some structural genes among the putative satellite pink clade phages may indicate the limited presence of cryptic, as well as satellite prophages.

### Distinct clustering of streptococcal phage by shared protein clusters

Shared protein clusters between *Streptococcus* phage sequences were analysed using vContact2 and visualized using Cytoscape (Fig. 6). Eleven prophages were identified as singletons by vContact2 and excluded from the cluster network. Two prophage clusters, I and II, comprised most prophage sequences (1,690/1,833; 92.20%). Cluster I typically contained larger genome, higher G+C content phages from both the current study and the VHDB (Table 2), whereas Cluster II comprised smaller genome, lower G+C content satellite phages from the current study and Javan *et al*. [15]. These clusters broadly correlated to the top and bottom clades of the ViPTreeGen proteomic alignment (Fig. 5), further supporting the division between full-length and satellite prophages. Clusters I, II and III represented the majority (1,802/1,833; 98.30%) of prophages in the dataset (Table 2). Each of these clusters contained phages from multiple streptococcal species, highlighting the sharing of protein clusters between different phages from different *Streptococcus* species (Table 3). Cluster I, containing full-length phages, showed many shared protein clusters between *S. uberis* prophages and phages from other streptococcal species, whereas the sharing of protein clusters was not as common in Cluster II, particularly between the *S. uberis* satellite prophages and phages from other hosts. These data confirm the division between putative full-length and satellite prophages.

**Fig. 6.**
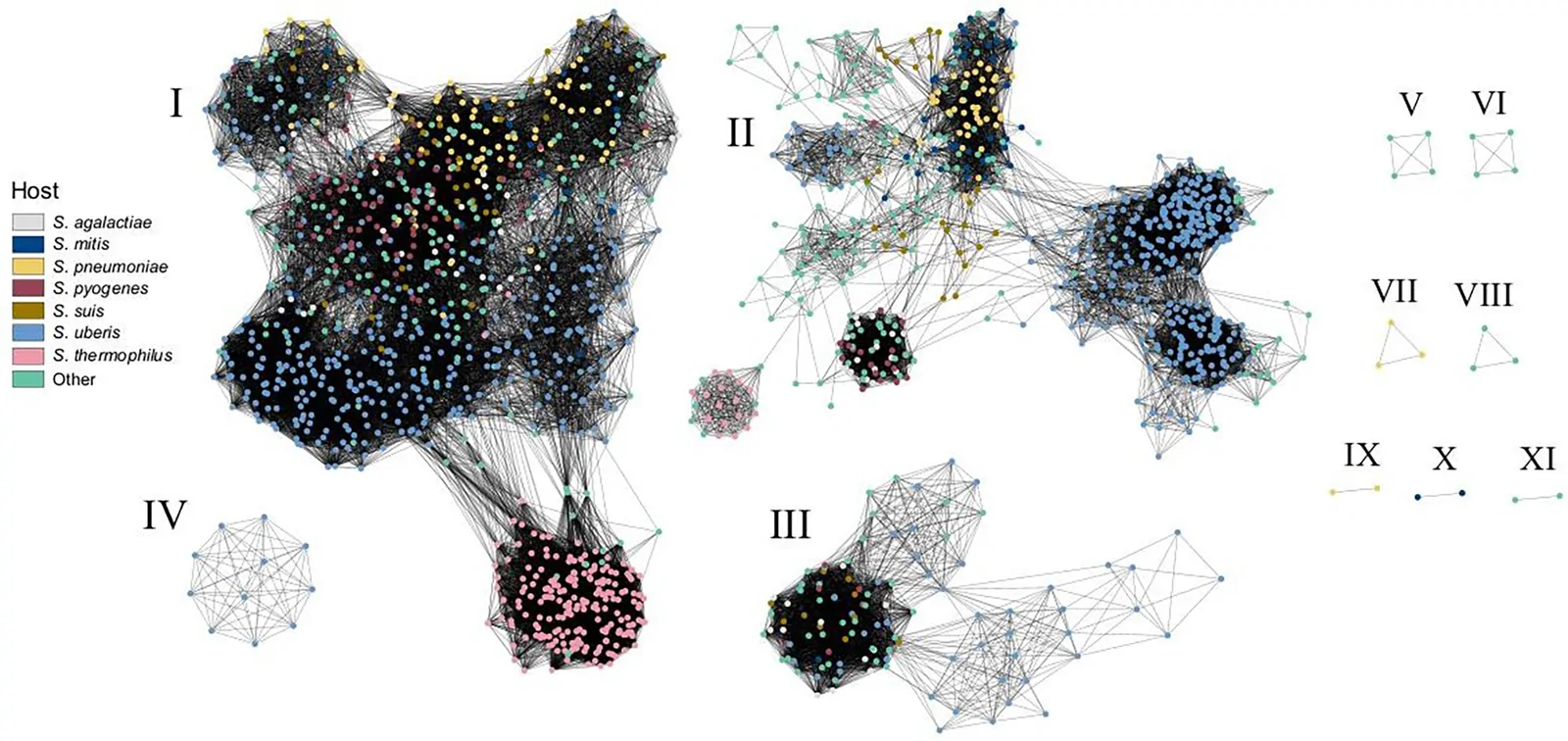
vContact2 protein cluster map of study *S. uberis* prophages (*n*=763), Javan *et al.* [15] satellite prophages (*n*=341) and VHDB streptococcal phages (*n*=729) coloured by the host bacteria species. Clusters were numbered I–XI. The seven most common host species were assigned colours, and the remaining were classed as ‘other’. Three study *S. uberis* prophages, seven Javan *et al.* [15] satellites and one VHDB streptococcal phage were excluded from clustering by vContact2. Network was visualized in Cytoscape.

**Table 2.** Summary information for vContact2 clustering of *S. uberis* prophages (*n*=763), satellite prophages (*n*=341) and VHDB bacteriophages (*n*=729). Mean average genome length (kbp) and genomic G+C content are shown for each cluster. All values are rounded to two decimal places, and values of 0 are shown as ‘–’

Cluster	Count	This study	Satellite	VHDB	Genome length (kbp)	G+C content (mol%)
I	1012	368 (36.36%)	–	644 (63.64%)	35.63	38.07
II	678	340 (50.15%)	334 (49.26%)	4 (0.59%)	14.67	34.87
III	112	44 (39.29%)	–	68 (60.71%)	42.73	40.05
IV	11	11 (100%)	–	–	11	30.07
V	4	–	–	4 (100%)	31.28	39.16
VI	4	–	–	4 (100%)	16.59	34.11
VII	3	–	3 (100%)	–	8.43	31.85
VIII	3	–	–	3 (100%)	19.48	38.73
IX	2	–	2 (100%)	–	8.73	31.95
X	2	–	–	2 (100%)	14.69	33.87
XI	2	–	–	2 (100%)	56.29	41.3

**Table 3.** Counts of host species in vContact2 clustering of *S. uberis* prophages (*n*=763), satellite prophages (*n*=341) and VHDB bacteriophages (*n*=729). Percentages for the row total are shown to two decimal places. All values of 0 are shown as ‘–’

Cluster	Count	*S. uberis*	*S. thermophilus*	*S. pneumoniae*	*S. pyogenes*	*S. suis*	*S. agalactiae*	*S. mitis*	Other
I	1012	375 (37.06%)	195 (19.27%)	87 (8.6%)	78 (7.71%)	39 (3.85%)	32 (3.16%)	20 (1.98%)	186 (18.38%)
II	678	351 (51.77%)	16 (2.36%)	44 (6.49%)	21 (3.1%)	25 (3.69%)	19 (2.8%)	35 (5.16%)	167 (24.63%)
III	112	46 (41.07%)	–	–	6 (5.36%)	10 (8.93%)	12 (10.71%)	1 (0.89%)	37 (33.04%)
IV	11	11 (100%)	–	–	–	–	–	–	–
V	4	–	–	–	–	–	–	–	4 (100%)
VI	4	–	–	–	–	–	–	–	4 (100%)
VII	3	–	–	–	–	–	–	–	3 (100%)
VIII	3	–	–	3 (100%)	–	–	–	–	–
IX	2	–	–	–	–	–	–	–	2 (100%)
X	2	–	–	–	–	–	–	2 (100%)	–
XI	2	–	–	2 (100%)	–	–	–	–	–

## Discussion

This study conducted the largest identification and analysis of prophages in *S. uberis* genomes to date. We reported an average of 1.16 prophages per isolate, whereas previous studies have reported a range from 0.37 to 2.4 prophages per isolate (0.37 [18], 0.54 [21], 0.58 [15], 1.16 [20] and 2.4 [19]). However, many of these studies investigated only small numbers of isolates, making them unrepresentative of the broader and highly diverse *S. uberis* population and with minimal characterization of the prophages identified.

Due to the relative lack of known *S. uberis* phages, PhiSpy was used for prophage identification, as it does not rely on sequence comparison with phage genomes [34]. CheckV was used as a quality filter for putative prophage sequences to reduce any misidentification of phages by PhiSpy [35]. However, an important limitation of the study is the use of short-read assemblies, and it is likely that prophages were missed due to them overlapping contig breaks or being present regions that did not assemble well [48]. Indeed, the apparent lower prevalence of prophage among USA isolates might be a function of the poorer quality assemblies of their genomes.

We have identified two phylogenetically distinct clades within the *S. uberis* prophage population. These clades are also distinct in terms of their genome length, G+C content and the presence of structural, endolysin- and holin-encoding genes. Their representative features are consistent with these two clades representing full-length and satellite *S. uberis* prophages. A shorter genome length and the absence of structural, endolysin- and holin-encoding genes are often features of satellite phages. The difference in G+C content between putative full-length and satellite prophages seen here likely relates to the loss of structural genes. These are often enriched in regions of high G+C content, and their loss in satellite prophages will have a disproportionate impact on the overall G+C content of the prophage [49].

It can be difficult to distinguish satellite prophages from cryptic prophages, which are the remnants of full-length prophages. However, the phylogenetic distinction, lack of shared genes and limited protein clustering between the two prophage clades support their genetic distinction from each other and the likelihood that the putative satellite prophages are not, in the main, cryptic versions of the identified full-length prophages.

Phylogenetic and protein cluster mapping of a genus-wide analysis of *Streptococcus* phages showed the same contrast between putative full-length and satellite prophages. The clustering of our putative satellite prophages with previously identified satellite prophages supports our designation of these as satellite prophages [15]. Our analysis with *S. uberis* corroborates findings in other *Streptococcus* species showing that full-length and satellite prophages are distinct with separate evolutionary histories [15].

Five highly similar prophages were identified as being present in distinct *S. uberis* isolates, namely, vB_SubZ_Freddy, Nice, Field and Milk from the current study and Javan 620 from previous work [15]. The identification of these putative satellite prophages in geographically disparate host strains suggests a capacity for excision and replication [48]. However, they could instead have spread by recombination, and despite their relatively high SG score to one another, their genome size differs substantially (8.80 to 23.01 kbp). This may indicate gene gain/loss following transduction or an artefact of different prophage identification tools.

Despite the use of the PHROG bacteriophage annotation dataset, over 60% of the genes in the S. *uberis* prophage pangenome were annotated as hypothetical proteins (2,830/4,606; 61.44%). This proportion was even higher among satellite prophage genes, where 72.00% were annotated as hypothetical proteins (720/1,000). This finding highlights the poor functional annotation, as well as the potential genomic novelty of the *S. uberis* prophage pangenome. Many of these genes could be important in phage-bacterial interactions and mediate lysogenic conversion [49, 50]. Novel structural or lysin genes may have been annotated as hypothetical proteins confounding the classification of the green clade prophages as satellite prophages. However, their other features relative to full-length prophages are still congruent with their classification as satellite prophages (i.e. genome length, G+C content and lack of characterized structural, endolysin and holin genes).

The prevalence of and distinction between full-length and satellite prophages have been reported within streptococci [15]. While Javan *et al*. only analysed 13 *S*. *uberis* genomes, we demonstrated that both groups of prophages are prevalent throughout the species. Furthermore, we showed that satellite prophages across the genus are characterized by an absence of structural and endolysin genes. While helper phages have been reported within other Gram-positive cocci [51], this has not yet been demonstrated in streptococci to the best of our knowledge.

While temperate phages are undesirable in their native form for phage therapy, their lysins could be exploited as novel therapeutics. There is already a precedence for phage lysins active against *S. uberis*, such as PlySs2 [25], PlySs9 [25], Ply700 [26] and B30 [23]. We identified 259 endolysin annotations in our *S. uberis* prophages which could provide the basis of future investigations for novel antimicrobials. For instance, phage-derived endolysins, similarly to bacteriocins, may have potential as a teat dip to control mastitis pathogens [52].

## Conclusion

We have conducted the largest prophage screening and characterization in *S. uberis* to date. This has revealed a diverse and poorly characterized prophage population of putative full-length and satellite prophages. This provides a foundation for future investigation of the biological and evolutionary significance of prophages in this prominent veterinary pathogen.

## Supplementary material

10.1099/mgen.0.001849 Supplementary Material 1.

## Abbreviations

NCBI: National Center for Biotechnology Information
PHROG: prokaryotic virus remote homologous group
VHDB: Virus-Host Database
